# Leading in the Paradoxical World of Crises: How Leaders Navigate Through Crises

**DOI:** 10.1007/s41471-022-00147-7

**Published:** 2022-12-16

**Authors:** Charlotte Förster, Caroline Paparella, Stephanie Duchek, Wolfgang H. Güttel

**Affiliations:** 1 Faculty of Economics and Business Administration, Junior Professorship of European Management, TU Chemnitz, 09111 Chemnitz, Germany; 2 Academy for Continuing Education, TU Wien, 1040 Vienna, Austria; 3 Center for Responsible Research and Innovation, Fraunhofer Institute for Industrial Engineering IAO, 10623 Berlin, Germany; 4 Institute of Management Science, TU Wien, 1060 Vienna, Austria

**Keywords:** Leaders’ behavior, Leadership, Organizational crisis, Organizational resilience, Paradoxes, H12 Crisis Management

## Abstract

Living and operating in a global world, the risk for a global economic crisis has never been greater. As ongoing events, such as the COVID-19 pandemic, the recent war in Ukraine or the sharply increasing inflation have shown, organizations need to be highly resilient to persevere in a crisis-prone world. Even though we know that crises serve as a focal lens on leadership behavior and leaders play a crucial role in these scenarios, little is known as to how leaders handle an existence-threatening organizational crisis. Using an inductive analysis of 32 interviews on crisis leadership, we show that in the case of an acute crisis, leaders apply different paradoxical behaviors to cope effectively with the situation and navigate their organizations through these events. More specifically, our study contributes to existing literature by, first, showing that the distinctiveness of crises results from the fact that leaders are confronted with paradoxes that they can otherwise smoothly separate in terms of time or organization, second, revealing that the leader’s paradoxical behaviors as a respond are derived from their mindset to consciously recognize the contradictory demands of the crisis, and third, from their action in terms of a compressed situational leadership. By identifying six pairs of paradoxical behaviors, we demonstrate how leaders effectively deal with the unsolvable contradictions that arise from the crisis, and thus contribute to the organizations’ ability to cope with crises.

**Supplementary Information:**

The online version of this article (10.1007/s41471-022-00147-7) contains supplementary material, which is available to authorized users.

## Introduction

Reflecting on Apple’s near bankruptcy in 1997, Steve Jobs said, (professional) “Near-death experiences can help one see more clearly sometimes” (Schlender and Tetzeli 2016). Even if formulated in an extreme way, the world actually seems to have moved from one crisis to the next since 2020 first climaxing in the recent COVID-19 pandemic (Rouleau et al. 2020) and now in the war in Ukraine and the risk of an inflation spiral. Although not many crises during the last years were specifically defined as organizational crises, companies were often deeply affected by the ramifications of these incidents.

Based on a fundamental definition of resilience as the extent or ability of a system to tolerate, manage and persist disturbance (Gilly et al. 2014; Gunderson and Holling 2001; Limnios et al. 2014), organizational resilience can be defined as ability of an organization to anticipate, cope with (or respond to), recover and learn from adversity in terms of the organizations ability to adapt (e.g., Duchek 2014, 2020; Hepfer and Lawrence 2022). Highlighted by the COVID-19 pandemic, dealing with unexpected events can be considered as a fundamental challenge in daily organizational life (Linnenluecke 2017), whereas resilience seems to be the capacity for organizations to meet the challenges that arise from these turbulent environments (Fietz et al. 2021; Hillmann et al. 2018; Lengnick-Hall et al. 2011).

Therefore, researchers as well as practitioners are interested in how organizations cope effectively with uncertainty and become more resilient. Using the Mann Gulch wildfire disaster of 1949 as an example, Weick impressively outlined the dramatic consequences of a leadership failure with the death of 13 firefighters in 1993. Even though we know that leaders have a crucial role in an organizational crisis (e.g., Pearson and Clair 1998; Weick 1993) and individual resources are vital to developing organizational resilience (Lengnick-Hall et al. 2011; Riolli and Savicki 2003; Horne and Orr 1998; Mallak 1998; McCann and Selsky 2012; Shin et al. 2012), researchers have yet to examine the relationship of leaders and organizational resilience in greater detail (e.g., Williams et al. 2017). Acknowledging this research gap, we examine how leaders handle existence-threatening organizational crises, and therefore navigate their organizations through these events.

Through the inductive analysis of 32 in-depth interviews and by focusing on the leaders’ role in a major organizational crisis, we show that deriving from both the leader’s mindset to consciously recognize the contradictory demands of the crisis and the leader’s action in terms of a compressed situational leadership, leaders apply different paradoxical behaviors to cope effectively with the situation and navigate their organizations through crisis.

Drawing from this analysis, we offer three main contributions to the existing research on crisis management (e.g., Pearson and Clair 1998), paradoxes (e.g., Smith 2014; Smith and Lewis 2011; Smith and Tushman 2005) and organizational resilience (e.g., Duchek 2020; Fietz et al. 2021; Williams et al. 2017). First, we outline that crises are highly paradoxical situations where contradictions can hardly be solved by making immediate decisions between opposing choices. Therefore, we show that paradoxes are not only important when it comes to organizational change (e.g., Carmine et al. 2021; Jay 2013; Luscher and Lewis 2008; Smith and Tracey 2016) but also when organizational crises emerge (Giustiniano et al. 2020). Second, in line with Lewis and Smith (2022, p. 18) who recently wrote “navigating paradox is paradoxical”, our findings demonstrate that in order to deal with the challenges arising from crisis, leaders need to consciously recognize these paradoxes as well as they need to align their behaviors. Therefore, navigating through crisis requires the leaders both to adapt their mindsets as well as their actions (Lewis and Smith 2022), which is exactly what makes crises so challenging for leaders.

Third, we argue that leaders cope with the crisis by consciously recognizing the contradictory demands of the crisis and behaving appropriately in terms of paradoxical leadership behaviors. In doing so, we contribute to the literature on organizational resilience by elucidating the role of the leader in existence-threatening organizational crisis. Even though some researchers have discussed the role of individuals in organizational resilience (Mallak 1998; Horne and Orr 1998; Fietz et al. 2021), the role of leaders in the development of organizational resilience has been particularly neglected from an empirical point of view (Van der Vegt et al. 2015; Williams et al. 2017). With a specific focus on the “cognitive and behavior attributes that facilitate resilience” (Williams et al. 2017, p. 752), we argue that leaders’ paradoxical behaviors help leaders to navigate through crisis, and thus foster organizational resilience.

## Theoretical Background

### Organizational Crisis and Resilience

Living and operating in a highly globalized and intertwined world (Li 2020), the risk of organizational crisis has never been greater. Organizational crisis is defined as “an event perceived by leaders and stakeholders as highly salient, unexpected, and potentially disruptive” (Bundy et al. 2017, p. 1662), whereby salience can be more narrowly defined as “the perceived significance of the impact” and “the perceived urgency of the response” (Wu et al. 2021, p. 2). Organizational crisis can have different dimensions, ranging from extortion, bribery, and product tampering to natural disasters that destroy organizational property or, even worse, kill organizational members (Pearson and Clair 1998). As recent disasters, such as the financial crisis in 2008 and the current COVID-19 pandemic, have shown us, the risk of organizational crisis is significant, especially due to globalization and internalization (Li 2020; Tourish 2020). Therefore, organizations need to be prepared for any kind of organizational emergency.

In previous resilience research, three perspectives on organizational resilience can be distinguished:

1. resilience as the ability to resist disturbances or recover after adverse situations (e.g., Horne and Orr 1998);
2. resilience as the ability to use crises for advancing organizational processes and developing new capabilities (e.g., Lengnick-Hall et al. 2011), and
3. resilience as the ability to anticipate and prepare for future crises (e.g., Somers 2009). Newer research agrees that resilience is a combination of these different perspectives (Bhamra et al. 2011; Duchek 2020). We follow this assumption and define organizational resilience as the ability of an organization to anticipate, cope effectively with, and learn from crises (e.g., Duchek 2014, 2020; Hepfer and Lawrence 2022; Limnios et al. 2014; Ortiz-de-Mandojana and Bansal 2016).

Further, considering resilience as a multilevel construct (e.g., Hartmann et al. 2020) implies that it is strongly interrelated with different levels of an organization. This means that resilience at the organizational level largely depends on the resilience of teams and individual members within the organization and vice versa. Therefore, to develop organizational resilience, individual resources are particularly important (Lengnick-Hall et al. 2011; Riolli and Savicki 2003; Horne and Orr 1998; Mallak 1998; McCann and Selsky 2012; Shin et al. 2012), especially with a view toward the upper echelon of the organization (Carmeli et al. 2013). Acknowledging the importance of individual resources for organizational resilience, particularly with reference to the role of the leader, the next section shows what we know about this relationship and how our study can contribute to this important research field.

### The Role of Leaders in Organizational Crisis and Resilience

Living and operating in a world where crises are omnipresent requires leaders to know how to steer their companies through any conceivable situation. Drawn from these circumstances, leaders are increasingly characterized by how they deal with adversity and whether they can learn from trying circumstances (Bennis and Thomas 2002). Since crisis are characterized by both significance and urgency, leaders’ decision-making during crisis is characterized by uncertainty, risk, and time pressure (Wu et al. 2021). However, crises do not only threat an organization, they also can represent “turning points for positive changes” (Wu et al. 2021, p. 3), and thus for growth and organizational resilience (e.g., Lengnick-Hall and Beck 2009) but only “when they are managed well” (Wu et al. 2021, p. 3). Organizational crisis therefore requires leaders to turn crisis into opportunities for growth and resilience (e.g., James and Wooten 2005), and not only to minimize “potential disruption” (Wu et al. 2021, p. 3). Thus, recognizing the role of leaders in organizational crises (Pearson and Clair 1998; Milburn et al. 1983; Roberts and Bea 2001; Williams et al. 2017), the question arises as to the leaders’ influence in the course of the crisis. In this regard, previous research in this context has recognized the leaders’ influence on their employees’ resilience (e.g., Avey et al. 2011; Gooty et al. 2009; Walumbwa et al. 2010; Harland et al. 2005; Rego et al. 2012). Moreover, researchers demonstrated that CEO greed (Sajko et al. 2021) and narcissism (Buyl et al. 2019) negatively impact organizational resilience. On a theoretical level, Samba et al. (2017) as well as Norman et al. (2005) each developed a model depicting the potential influence of the leader on organizational resilience. Norman et al. (2005) theoretically explained how the leaders’ state of hope influences not only their own but also their followers’ resiliency, which leads to organizational resilience affecting the long-term success. Samba et al. (2017) focused on the impact of positive leadership on organizational resilience by describing how positive leadership creates structural conditions in terms of a positive infrastructure that fuels the process of organizational resilience. Nonetheless, the relationship between the leader and their organizations in terms of resilience remains vague, which means that we do not know how the leaders deal with the paradoxes arising during crises, and how this might help them to navigate their organizations through crises.

Although leaders play a significant role in crises, so far neither the causes why crises are experienced as so stressful nor their concrete solution or survival strategies to ensure the survival of their organizations have been investigated. Referring to the crucial role leadership plays in the context of organizational resilience (Samba et al. 2017, Norman et al. 2005; Sutcliffe et al. 2017; Williams et al. 2017), our study aims to examin how leaders handle existence-threatening organizational crises, and therefore navigate their organizations through these events.

## Methodology

### Research Approach

Due to the particularity of the crisis situation and the limited previous research on the leaders’ role in the pursuit of a resilient organizational response to crises (e.g., Williams et al. 2017), we considered a qualitative research approach to be appropriate for our study, especially since our aim is to reveal new concepts instead of confirming old ones (Wickert and De Bakker 2018).

### Sample and Data Collection

Our study was part of a larger research project on crisis leadership, which gave us access to leaders of organizations that had experienced a major crisis. Data collection included 40 narrative interviews with leaders of various organizations facing severe crises over a period of 18 months. Our empirical study focuses solely on organizations that are faced with existence-threatening crises (e.g., loss of key customers, sales collapse, financial crises). Owing to our study restrictions, we only selected those interviews in which the leaders classified the experienced crisis as major and existence-threatening for the organization. This left us with 32 interviews for data analysis, in which various interviewed leaders stated that they faced the most difficult period in their lives. The interviewees were employed in firms of different sizes and based in various business sectors, i.e., finance, insurance, mechanical and electrical engineering, education, technology development, information technology (IT), energy, construction and plastic industry, and aircraft construction. For more details, see Table 1.

**Table 1:** Interview Data

Interview	Company	Industry	Position	Type of crisis
I1	C1	Construction	Head of Sales	Financial crisis 2008
I2	C2	Construction	Head of Human Resources	Financial crisis 2008
I3	C1	Construction	IT Project Manager	Financial crisis 2008
I4	C3	Bank	Head of Human Resources	Corruption
I5	C4	Bank	Head of Retail	Corruption
I6	C5	Technology	Controller	Financial crisis 2008
I7	C3	Bank	Project Manager	Corruption
I8	C3	Bank	Head of Software	Corruption
I9	C6	Automotive	Head of Product Development	Financial crisis 2008
I10	C7	Energy	Risk Manager	Financial crisis 2008
I11	C8	Bank	Head of Legal Services	Financial crisis 2008
I12	C9	Fiber production	Head of Human Resources	Drop in prices
I13	C10	Aviation / Bank	Chief Executive Manager	Nationalization
I14	C11	Energy	Human Resource Manager	Oil disaster
I15	C12	Consumer goods market	Chief Operating Officer	Market slump
I16	C13	Construction	Manager Large Scale Products	Financial crisis 2008
I17	C15	Agricultural equipment	Managing Director	Merger
I18	C15	Agricultural equipment	Area Manager	Merger
I19	C15	Agricultural equipment	Head of Sales	Merger
I20	C15	Agricultural equipment	Head of Central Services	Merger
I21	C15	Agricultural equipment	Head of Change	Merger
I22	C15	Agricultural equipment	Head of Department	Merger
I23	C16	Mechanical engineering	Chief Executive Officer	Financial crisis 2008
I24	C16	Mechanical engineering	B‑1 Manager	Financial crisis 2008
I25	C17	Telecommunication	Area Manager	Drop in sales
I26	C18	Medical Devices	Internal Manager	Product recall
I27	C19	Financial Services	Human Resources Manager	Financial crisis 2008
I28	C20	Public Law	Project Manager	Software crash
I29	C21	Consulting	Project Manager	Financial crisis 2008
I30	C22	Construction	Founder/Manager	Financial crisis 2008
I31	C23	Software	Head of Internal Services	Financial crisis 2008
I32	C24	Engineering	Chief Executive Manager	Financial crisis 2008

In contrast to the heterogeneity in terms of company sizes and business sectors, all of our interviewed leaders were key persons responsible for the handling and coordination of the respective organizational crisis. In addition to this deep involvement in the respective crisis, all leaders held high positions that covered a large area of responsibility. Along this line, one of the participants stated *“(…) that means that I manage projects with full responsibility for costs, budget responsibility (…)” *(I29, consulting, project manager, financial crisis 2008), whereby another project leader described *“(…) that was a staff position on the executive board (…) I managed strategic projects (…) ongoing monitoring and reporting of strategic projects”* (I7, project manager, bank, corruption).

To gain profound and comprehensive insights, the participants were encouraged to explicitly refer to the experienced organizational crisis in question and communicate their stories with all the details (e.g., behaviors, feelings, context factors). By mentally going through their crises, the leaders were able to remember their behavior more precisely (“critical incident technique”; Flanagan 1954). With the help of this technique, we could gather different patterns of behaviors in various incidents and analyze them in-depth. For instance, when we interviewed a leader from an organization (a global leader in the market for sustainable botanic cellulose fibers) who encountered grievous problems by facing a radical drop in the worldwide cotton price of 40%, we aligned our questions with this crisis to better understand how the leaders in charge encompassed the challenge they faced and how they handled the crisis effectively. For this purpose, we, for example, asked, “How did you feel when the cotton price fell?”

On average, our interviews lasted for 48 min each, ranging from 13–100 min. All interviews were recorded and transcribed afterwards. To protect anonymity, we removed all identifying information from the text (e.g., names of interviewees, firms, places).

### Data Analysis

Since our study was predominately inductive, the Gioia methodology (Gioia et al. 2012) appeared to be adequate to analyze our transcribed interview material. It provided the basis for delineating themes and clustering single codes to aggregated dimensions. Coding was performed with MAXQDA 2020.

During the first step (first-order analysis), we read the interview transcripts several times in order to group our participants’ experiences into broader categories (Wickert and De Bakker 2018). By using the words, phrases, terms, and labels offered by our participants, we kept an informant-centric perspective, as required in the first-order analysis (Gioia et al. 2012; Van Maanen 1979). By seeking similarities and differences among these categories, we reduced the number of categories to a manageable number, namely 29 first-order themes (Strauss and Corbin 1998; Gioia et al. 2012).

**Fig. 1:**
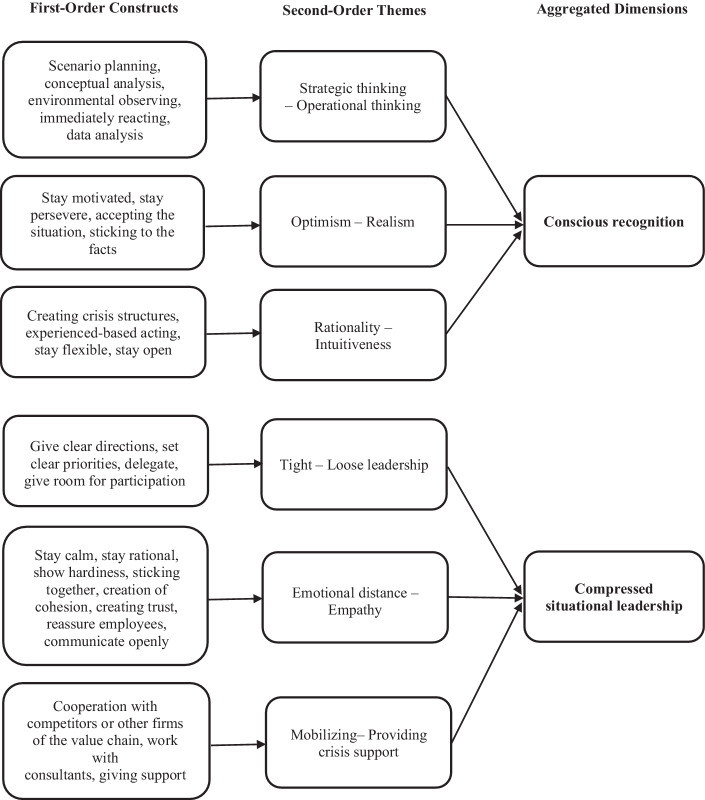
Data Structure

During the second step (second-order analysis), we switched to a researcher-centric perspective by consulting the relevant literature (Gioia et al. 2012; Van Maanen 1979). By repeatedly reviewing the data and the relevant literature, we were able to identify appropriate theoretical labels for our identified concepts. In sum, we identified six pairs of paradoxical leaders’ behaviors: (1) strategic thinking—operational thinking, (2) optimism—realism, (3) rationality—intuitiveness, (4) tight—loose leadership, (5) emotional distance—empathy and (6) mobilizing support—providing support. After reaching a sufficient number of themes and concepts, the last step of the data analysis consisted of building aggregated dimensions, thus leading to “theoretical saturation” (Gioia et al. 2012, p. 20; Glaser and Strauss 1967). While searching for suitable aggregated dimensions to which our second-order concepts could be assigned, we found that our leaders’ behavior resulted from two dimensions, namely the leaders’ mindset, labeled as conscious recognition, and the leaders’ actions, labeled as compressed situational leadership. The final data structure is illustrated in Fig. 1.

## Findings

Resulting from our explorative research approach, we found that the leaders’ behavior during major crises was not straightforward but somehow paradoxical. Paradoxes can be defined as contradictory but still interrelated; they exist simultaneously, thus leading to a certain tension (Smith and Lewis 2011, Lewis and Smith 2022; Zheng et al. 2018). In the case of our leaders, this means that they have to apply contradictory behaviors at the same time, which implies an additional challenge for them. Even though we acknowledge that successfully navigating one’s organization through crisis also depends on how the leader behaved before and after the crisis occurred (see also Williams et al. 2017; James and Wooten 2010), findings from our study show that in line with recent literature (Giustiniano et al. 2020) paradoxes and contradictions increase sustainably during crisis, thus requiring the leader to apply opposing behaviors. By handling these paradoxes successfully, leaders were able to navigate their organizations through crisis. In the following sections, we provide insights into the paradoxical behaviors that leaders applied when confronted with a crisis that threatened the existence of their organizations.

### Conscious Recognition

First, our leaders needed to recognize that they operate in an environment where paradoxes and contradictions are neither solvable nor avoidable. We labeled this phenomenon as “conscious recognition.” Given the mindset that a crisis is a highly contradictory situation, our participants adapted their behavior by relying heavily on paradoxical leadership behaviors to meet these opposing requirements. By combining extreme positions, our leaders were able to balance the paradoxical demands of the crisis. More precisely, they showed the following three combinations:

1. strategic vs. operational thinking,
2. optimism vs. realism, and
3. rationality vs. intuitiveness.

#### Strategic Thinking Vs. Operational Thinking

First, strategic thinking refers to the longer-term perspective, describing a way of thinking that is rather abstract and often less tangible. Operational thinking refers to the here and now, which is needed to handle the current situation and requires permanent attention. Our interviews showed that leaders need to apply both modes simultaneously in order to get their organizations out of crisis mode. More precisely, at the strategic level, our participants engaged in scenario planning and conceptual thinking, while, at the operational level, they observed current developments and took immediate actions.

*Scenario planning* appeared to the leaders, more than anything else, as crucial in outlining various developments in the unfolding crisis; thus, it was a major component of crisis leadership. By developing various scenarios, the participants thought about the possibility of future events and the unfolding crisis to confront unfortunate incidents and developments.

“And then you become more creative and say, if it doesn’t work that way, maybe it can be done differently. [You think] about things that you haven’t thought of because you haven’t had the need to.” (I18, area manager, agricultural equipment, merger)

With *conceptual analysis*, our leaders stressed the importance of independent and abstract analyses of new ideas or solutions for the future of their organization. Disconnected from the operational context, leaders used time spent during their daily routines, such as driving their car, being at home, or taking a shower, to put aside commonly accepted beliefs or constraints regarding their organization’s future. They looked for viable alternatives that were not obviously related or easily identified to address underlying problems. Some leaders deliberately set time aside to critically reflect on external developments. One of the participants commuted twice a week and used the time to think:

“And then I projected, 800 h; these are roughly 100 working days you spend in the car. A lot of time to think.” (I15, chief operating officer, consumer goods market, 2008 financial crisis)

At an operational level, participants viewed the *observation of current developments* as essential, describing it as a key duty for leaders. By observing other markets, market leaders, and competitors, the leaders sensed early indicators for possible market eruptions. Along this line, the participants reported that they extensively engaged in *data analysis*, such as performance and revenue streams, while also relying on indicators that had been established for the company or transmitted by the parent company.

“We observed the market very closely, the daily news; we monitored the automotive and aircraft industries very closely. They are an indicator, that is how it is.” (I24, B‑1 manager, engineering industry, 2008 financial crisis)

In this context, the participants underscored the importance of *immediate reactions*, which means that aside from observing, analyzing, and engaging in mental activities, the leader must translate the resulting evaluations into prompt actions.

“And then I was called (…) We, all those responsible, went straight to the data center and first tried to find out where the problem occurred.” (I26, internal manager, product recall).

#### Optimism Vs. Realism

Second, when confronted with the crisis, our leaders had to show optimism but simultaneously needed to keep a realistic view. While they did as much as possible to obtain the outcomes they wanted, cautiously hoping for favorable results, they also needed to accept the reality of the ongoing situation. This meant that they could not disregard negative information about the future of their companies, but rather had to report events as they were. We labeled this paradox as “optimism vs. realism.”

By maintaining a positive view about the future and believing in the company’s strength, our leaders *stayed motivated*. Small successes gave them hope to continue, take on challenges, and grow with the task.

“I think optimism is always very important. If we complain and say, ‘For God’s sake!’ and everything, it can only get worse.” (I9, head of product development, automotive, 2008 financial crisis)

Even though the rigor of the crisis pushed several leaders to their limits, they were convinced that they wanted to continue the journey and respond to the situation with increased persistence. Our leaders worked extremely hard and were reluctant to give up, especially because of their employees. *Perseverance* was vital for these leaders.

“You always have to believe that there is a solution. The moment you no longer believe in it, you have lost.” (I13, chief executive manager, aviation/bank, nationalization)

Despite this positive approach, the interviewees also underscored that they needed to *accept the situation* and confront what was currently happening in the organization. In addition to accepting the situation, the leaders also made clear that *sticking to the facts* helped them to come up with reasonable arguments and keep discussions from becoming unconstructive.

“In a crisis, you rely much more on what is written […]. Emails suddenly become very important.” (I3, IT project manager, construction industry, 2008 financial crisis)

#### Rationality Vs. Intuitiveness

Third, the participants usually relied on existing structures and knowledge to manage crisis situations. This meant that they built on previously gained experiences with challenging business situations to react to these situations and make solid decisions. Nonetheless, the leaders were also aware that they had to divert from previous solutions from time to time, since they could also be misleading. Therefore, decision-making in these emergency situations can be considered as “rationality vs. intuitiveness.” Leaders not only have to create crisis structures and act based on their experiences, but they also need to remain open and flexible.

Various leaders stressed the importance of crisis structures in terms of, for instance, specific crisis units or crisis leadership systems. Regardless of whether these structures were established before or during the crisis, *creating crisis structures* helped the organizations to progress and quickly return to normal. Even when the crisis was over, solid crisis structures were seen as a competitive advantage from the leaders’ perspective.

“I think the organizational structure is very important. I think the most important thing is to have a crisis team and to recognize crisis leadership as a separate discipline in the company.” (I26, internal manager, medical devices, product recall)

In addition, experiences were mentioned as a determining factor during the crisis, and these provided significant value for the interviewed leaders. Fundamental decisions were often made based on intuition, which again was derived from experiences, especially when leaders had to react quickly. Hence, *experienced-based acting* by the leader was of great importance during the crisis.

“That means after five years […], I was much more used to [crisis situations], whereas other people were terrified of [them].” (I13, chief executive manager, aviation, nationalization)

In contrast to this experience-based, routinized, and proven behavior, the participants also stressed a more flexible, open approach. In this context, they demonstrated that *staying flexible* regarding new ideas and unusual solutions was necessary when confronted with sudden threats. Furthermore, *staying open* to different perspectives and opinions was mentioned as being equally important when confronted with an unknown future.

“Then you actually throw all the traditional rules overboard. That’s the only way you can survive.” (I13, chief executive manager, aviation/bank, nationalization)

### Compressed Situational Leadership

In addition to the leaders’ mindset of conscious recognition, we also found that leaders dealt with occurring contradictory demands in crises using a specific leadership strategy, which we labeled *compressed situational leadership*. This term denotes the specific aspect of time in crisis situations, indicating that leadership at such times is much more pronounced and instantaneous or simply compressed. Thus, by applying different leadership styles in crisis situations, the leaders laid the foundation for what Weick (1993, p. 642) called “[r]espectful interaction.” More precisely, leaders showed the following three combinations:

1. tight vs. loose leadership,
2. emotional distance vs. empathy, and
3. mobilizing vs. providing crisis support.

#### Tight Vs. Loose Leadership

In order to handle critical situations effectively, leaders needed to give clear directions and set priorities. However, they also need to simultaneously delegate and make room for participation, thus allowing new solutions or hidden paths to be discovered. We summarized these behaviors under the paradox “tight vs. loose leadership.”

*Giving clear directions* means that leaders need to provide guidance to make sure that their employees are heading in the right direction and staying motivated.

“The most important thing is to provide employees with a clear goal and to explain why they are in a stressful situation and why it is important that they act in a certain way.” (I8, head of software, bank, corruption)

Along with a clear direction, participants also stressed the importance of *setting clear priorities*, including well-defined responsibilities. For instance, the leaders largely agreed to primarily deal with strategic issues, thus avoiding involvement in operational decisions.

“Then you have to set very clear priorities, to say very clearly, ‘I’ll do that now, and I can’t do the other thing now.” (I8, head of software, bank, corruption).

At the same time, our leaders also need to be aware of when and what to delegate as well as where to provide room for participation. In terms of *delegation*, the interviewees emphasized the importance of distributing authority and temporarily shifting responsibilities from the leadership to the team level. By delegating decisions to their teams or employees, leaders successfully relocated several aspects of the organization’s crisis response. This involved granting the authority to make and implement decisions without having to gain approval. In addition, leaders encouraged their teams to present their own ideas by *giving room for participation*, which helped gain new perspectives and solutions.

“So, for example, I included people that I absolutely trust. After two weeks, we got along so well that I knew I didn’t need to set a framework.” (I13, chief executive manager, aviation/bank, nationalization)

#### Emotional Distance Vs. Empathy

Showing empathy while staying emotionally distanced was mentioned as a key factor by the participants to persist through difficult events. United under the paradox “emotional distance vs. empathy,” the leaders explained that during crises, staying calm and rational and showing robustness is equally important as working together, creating cohesion and trust among their employees, and communicating openly to reassure their teams. *Staying calm* was outlined as a vital factor by the leaders in making good decisions and conducting an in-depth analysis of the situation. Furthermore, they reported that staying calm generally helped them to avoid stress.

“[It is important] that you do not run around like a startled bunny in your daily work and constantly have beads of sweat on your forehead, but that you still face this crisis and your daily business with a smile and with a certain calmness.” (I22, head of department, agricultural equipment, merger)

Moreover, to cope with the crisis situation while serving as a role model for their employees, the participants underscored the need to *stay rational* by suppressing the emotional aspects of the crisis. In line with this, the participants also reported that *showing hardiness* helped them to keep control by acting strictly and consequentially and even by increasing pressure in front for their employees.

“You just have to be able to concentrate on, let’s say, the job and just switch off this (…) this soft, interpersonal relationship.” (I7, project manager, bank, corruption)

In contrast to this calm, rational, and even hardy approach, the leaders also mentioned that *sticking together *with their team was highly important to them. By doing so, the participants reported a strong sense of solidarity and unity, and this behavior also enabled them to face the situation together. In this context, the interviewees told us that relying on their employees to run day-to-day operations made the *creation of a certain degree of team cohesion* inevitable. From the leaders’ perspective, a culture that was already characterized by such cohesion certainly favors working together in times of crisis.

“Whenever employees know each other well and have a way with each other at a personal level, then collaboration in daily operations runs smoothly.” (I3, IT project manager IT, construction, 2008 financial crisis)

The interviewees highlighted that *creating trust* by conveying reliability was crucial during the crisis. In this sense, the leaders emphasized the importance of transmitting trust and security to their employees, even though they did not know what to expect. Along these lines, participants also reported the need to *reassure their employees* by reducing fears about losing their jobs and an uncertain future. This requires a certain degree of empathy in contrast to the abovementioned emotional distance, which is also needed.

“What again is very important (…) is to give this security and this trust to the employees. But that only works if this topic of trust is already at a very good level in the company.” (I30, founder/manager, constructing software and recruitment, 2008 financial crisis)

Although creating trust was an important behavior mentioned by our leaders, they also stressed that *communicating openly* with their employees was essential for prevailing in a critical situation. By communicating transparently and, for instance, explaining why certain decisions were made, our participants helped to orient their employees and motivate them to persevere.

“We informed the employees extremely often. We kept holding conference calls for our 1000 sales employees.” (I15, chief operating officer, consumer goods market, market slump)

#### Mobilizing Vs. Providing Crisis Support

Finally, to promote rapid problem solving and perform under high-stress and chaotic conditions, the leaders needed to mobilize support through their networks—either inside or outside the organization, but also had to provide support to their own employees and colleagues. Since this paradox required the leaders to be both self- and other-oriented, we labeled this paradox as “mobilizing vs. providing crisis support.”

In terms of mobilizing support, our leaders relied on external cooperation with various institutions, competitors, as well as firms from their value chain, and involved consultants to effectively cope with the acute crisis. In more details, leaders reported of close relationships with, for instance, actors in media who provided support in terms of crisis communication. Furthermore, some leaders also reported long-term *cooperation with competitors or other firms of the value chain* that turned out to be crucial to the company’s survival.

“[It is important] to build a relationship with competitors, where you are not seen as a competitor but as a market participant (…) who has his core competencies in these areas but is also ready, if it is not his core competency, to pass service to the best possible company.” (I6, controller, technology, 2008 financial crisis)

In addition, the interviewees also *worked with consultants* to obtain an outside perspective to improve their decision-making. By including people from outside the organization, the leaders tried to eliminate their blind spots.

“We deliberately looked for people from outside [the company].” (I13, chief executive manager, aviation/bank, nationalization)

However, the leaders also needed to provide resources to both their colleagues and their employees. Thus, in order to navigate their organizations successfully through the crisis, the participants had to carefully manage their resources. While the leaders were highly dependent on the support of others, for example, as described above, external consultants and their own employees, leaders also had to provide resources in terms of support to others. Along this line, the leaders always tried to have an open door for the employees’ concerns. In this context, the participants made sure that coaching was not only available for the leaders but also for the employees and some of the leaders also initiated financial support programs for the employees that had to be laid off. By this means, mobilized support by others but also *gave support* to their employees with the aim of reducing their stress level during crisis.

“There is still an offer (…) that you can use leadership coaching for yourself. There is also the possibility [for the employees] to do anonymous coaching.” (I4, head of human resources, bank, corruption)

This section shows us that in the case of an acute crisis, leaders apply different paradoxical behaviors to cope effectively with the situation and navigate their organizations through the crisis. These behaviors are derived, on the one hand, from the leaders’ mindset to consciously recognize the contradictory demands of the crisis, and, on the other hand, form the leaders’ action in terms of a compressed situational leadership. In the next section, we will discuss these results in the context of current research and explore how this behavior might help leaders to build resilience in their organizations.

## Discussion

**Fig. 2:**
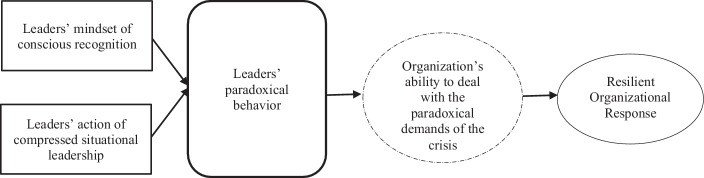
How paradoxical leadership behavior foster a resilient response during crisis

In line with Linnenluecke and Griffiths (2010, p. 478) who argue that “[a] significant challenge for organizational decision makers and leaders has always been to deal with unexpected changes in their organizations’ environments” (King 1995; Weick and Sutcliffe 2001), the leaders in our study show different paradoxical behaviors to cope effectively with the situation and navigate their organization through the crisis. More specifically, we argue that by increasing the ability of their organization to cope with the crisis, leaders might also foster a resilient organizational response to these existence-threatening crises (see Fig. 2). In the following, we therefore explain our contributions that, first, the leader’s perception of paradoxical demands in crisis is crucial, second, that the leaders’ mindset of conscious recognition is an important response and, third, the action of compressed situational leadership contributes to organizational resilience might help organization to build organizational resilience during crisis.

### Recognizing Paradoxes and the Leaders’ Mindset of Conscious Recognition

By knowingly recognizing that crises are highly paradoxical and contradictory situations that require the leader to act accordingly (Smith and Lewis 2011; Tabesh and Vera 2020), we show how leaders align their own behavior with these paradoxical demands by using opposing behavior. In line with Smith and Lewis (2022) who argue that navigating paradoxes require underlying beliefs and mindsets that enable “to cognitively hold two opposing forces at the same time” (Lewis and Smith 2022, p. 12), our data show that leaders have to consciously deal with emerging paradoxes in crisis. Thus, to handle organizational crises, leaders predominately engage in behaviors that are simultaneously strategic and operational. Not only do they prepare their organizations for the upcoming crisis, but they also manage to deal with the paradoxical requirements arising from the crisis. Previous literature indicates that activities such as understanding and observing the environment (Vogus and Sutcliffe 2007; Pinkse and Gasbarro 2019) and scenario planning (Hillmann et al. 2018) can increase organizational resilience (Fietz et al. 2021), whereby Mumford et al. (2007, p. 515) outline scanning, both of their internal (Ford and Gioia 2000; O’Connor 1998) as well as their external environment (Souitaris 2001), as the starting point in leader cognition “under conditions of crisis or change”. Scanning activities according to the authors encompass an “ongoing environmental monitoring” which itself is “a low cost, ongoing, activity” that not necessarily needs to trigger a leaders’ reaction (Mumford et al. 2007, p. 527).

In order to shed light on the role of the leader in existence-threatening organizational crisis, we demonstrate that paradoxes are not only important when it comes to organizational change (e.g., Carmine et al. 2021; Jay 2013; Luscher and Lewis 2008; Smith and Tracey 2016) but also when organizational crises emerge (Giustiniano et al. 2020). Leaders apply both strategic and operational behaviors to avoid suffering from what is known in existing research as the problem of myopic leadership tendencies. This means that leaders prefer short term over long term (Smith and Tushman 2005; Levinthal and March 1993) and focus more on quick wins. In addition, there are typically more operational than strategic issues, which tempts leaders even more to engage at the operational level, which considerably impacts decision-making during crisis (Boin et al. 2013). Even though most research was conducted under normal conditions when the organization was not in crisis (Smith 2014; Smith and Tushman 2005), this kind of problem probably exacerbates during a crisis because a quick win might appear even more tempting when a lot is at stake, presumably regardless of the long-term consequences. In this regard, Sajko et al. (2021, p. 957) demonstrated that for instance “greedy CEOs are more likely to exhibit myopic behaviors”, whereby organizations led by greedy CEOs are more likely to exhibit lower resilience. Resilience in this context was operationalized as the short-term losses as well as the recovery time after the 2008 global financial crisis (Sajko et al. 2021). Additionally, Fietz et al. (2021) recently implied that to increase organizational resilience, both a long-term as well as a short-term orientation is needed. The authors further indicate that a long-term orientation might be necessary to build a sustainable network and alliances (Borekci et al. 2014; Fietz et al. 2021; McCann and Selsky 2012), whereas short-term orientations are “related to action-orientation” (Fietz et al. 2021, p. 33; McCann and Selsky 2012), which is essential in an acute crisis.

We also found that instead of sticking to one position of either being extremely idealistic or extremely pessimistic, leaders remain optimistic during the crisis but do not lose their realistic perception, for instance, by sticking to the facts (Coutu 2002). In this way, they maintain courage and create a motivating atmosphere by passing this spirit on to their employees. At the same time, however, they do not close their eyes to reality and, therefore, they continue to make informed decisions. By keeping a realistic and optimistic view of the crisis (Coutu 2002), leaders are also more likely to “perceive crises as opportunity” (Brockner and James 2008, p. 94), one that includes change, growth, and eventually resilience (Sayegh et al. 2004; Brockner and James 2008; Golan 1978).

Furthermore, crises generally encompass a central conflict between time and accuracy. More specifically, crisis situations require rapid responses, whereby “information gathering (…) is a time-consuming activity” (Mumford et al. 2007, p. 532). In addition, and as the COVID-19 pandemic has shown us, information during crisis is highly ambiguous, comes from various sources, and can sometimes be misleading (e.g., Siebenhaar et al. 2020). Nonetheless, leaders’ decision-making in crisis often needs to be quick (Kerrissey and Edmondson 2020; Sayegh et al. 2004) or at least take place under perceived time pressure (e.g., Pearson and Clair 1998). Therefore, it is essential for leaders to rely on their experiences for decision-making in crisis. Sayegh et al. (2004) asserted that experiences are the foundation for emotional or intuitive leaders’ decision-making. However, as the current COVID-19 pandemic has vividly demonstrated, experiences can also be misleading, because what we learned in the past might not be suitable in the present (Foerster and Duchek 2022; Reeves et al. 2020). Therefore, it is essential for leaders during crisis to knowingly recognize that paradoxical situations require a behavior that is led by both a rational and an intuitive approach, for instance, by staying open and flexible. While “improvisation and bricolage” are known to be important aspects of resilience (Weick 1993, p. 638), our study results demonstrate that both improvisation as well as making informed and reasonable decisions are highly important when it comes to managing adversity (Best and Gooderham 2015; Lengnick-Hall et al. 2011; Tabesh and Vera 2020).

### Fostering Organizational Resilience Through Compressed Leadership Behavior

Leaders deal with contradictory demands in crisis by concentrating on priorities, making fast decisions, delegating, and allocating room for participation. They simultaneously use a tight and loose leadership strategy, which could also be described as “command and control” (Boin et al. 2013) on the one side and “persuasion” or “enabling leadership” (Boin et al. 2013; Nooteboom and Termeer 2013) on the other side. By doing so, they are able to give clear directions and set clear priorities while allowing scope for action through delegation. In this way, leaders could maintain control while also rationing their time, space, and energy to act quickly and directly address essential, high-level tasks. From an organizational perspective, both aspects are especially crucial in times of crisis (Boin et al. 2013).

Since organizational crises are inherently unpredictable (e.g., Bundy et al. 2017), leaders must always have sufficient room to maneuver and react accordingly. At the same time, losing direction would be inexcusable during a crisis. Therefore, both can be regarded as important to organizational resilience, especially during a crisis. By applying this tight and loose leadership behavior, leaders integrated elements of both a democratic and autocratic leadership style (Weick 1993). While Weick (1993) argued that leaders temporarily shift between both of these contradicting styles, we argue that, due to the contradictory conditions in crisis situations, leaders need to apply them both simultaneously. In addition, an organizational crisis is an “emotionally charged situation” (James and Wooten 2005, p. 142; Meisler et al. 2013), whereby emotional reactions might lead to “pessimism, defensiveness, feelings of trauma and betrayal, ignorance and grief” (Bundy et al. 2017, p. 1676; Kahn et al. 2013; Mitroff 2007; Roux-Dufort 2007; Vaaler and McNamara 2004). To conquer this kind of situation, the leaders need to be both emotionally distanced, for example, by staying calm and rational, and show empathy by creating trust and a certain degree of cohesion. These findings correspond in a certain way to Smith et al. (2020), who argued that leaders need to balance their analytical and emotional intelligence. By doing so, leaders are able to knowingly deal with these emotions and not lose their ability to behave rationally. They are also able to create an organizational atmosphere consisting of trust and cohesion which is important in building organizational resilience during crisis (Gittell et al. 2006; Mafabi et al. 2015).

Aspects of what we labelled as compressed leadership behavior might also be known from the situational leadership theory (SLT; Hersey and Blanchard 1988 [1982]; Norris and Vecchio 1992). Based on the development/maturity level of the follower, four different leadership styles, defined as a “combination of relationship-oriented behavior and task-oriented behavior”, are recommended by the authors (Norris and Vecchio 1992, p. 331; Blanchard et al. 1993). In contrast to the situational leadership theory, the leaders’ behavior of tight and loose leadership is not an either- or strategy but combines both dimensions in response to the paradoxical demands of the crisis.

By applying both leadership strategies, tight–loose leadership and emotional distance–empathy, our leaders lay the foundation for what Weick (1993, p. 642) called “[r]espectful interaction.” Respectful interaction results from intersubjectivity (Weick 1993; Wiley 1988), which is nurtured by trust, honesty, and self-respect (Campbell 1990; Weick 1993) and which seemed to be missing “in several well-documented disasters in which faulty interaction processes led to increased fear, diminished communication, and death” (Weick 1993, p. 643). By showing empathy and allowing room for participation, while simultaneously maintaining a clear line and a certain distance, leaders enable respectful interaction despite the contradictory demands of the crisis and, therefore, are able to foster organizational resilience during crisis.

Finally, navigating through crisis requires a wide range of support (e.g., Nichols et al. 2020), whereby networks and alliances can be highly important to build organizational resilience (Borekci et al. 2014; Fietz et al. 2021; McCann and Selsky 2012). Therefore, leaders look for support, for example, by working with consultants. However, providing support to employees is equally important, especially to create an atmosphere of trust. Thus, although support is important during crisis (e.g., Nichols et al. 2020), it is even more important that this is not a one-way street; rather, it requires reciprocity. Therefore, mobilizing but also providing support requires the leaders to manage his or her resources carefully, especially in times of crisis where both time and resource are scarce.

## Conclusion, Implications, and Limitations

This study focuses on understanding how leaders behave when the organization faces an existence-threatening crisis, and how this might help organizations in pursuing a resilient response. Defining organizational resilience more broadly as the organizations’ ability to handle crisis and grow through it (e.g., Gilly et al. 2014; Williams et al. 2017), we identified the leaders’ mindset of conscious recognition and their action of compressed situational leadership as crucial to the leaders’ ability to align their behaviors to the paradoxical demands of the crisis. In extension to Lewis and Smith (2022, p. 7) who describe paradoxes as involving “dualistic forces” that both opposing as well as reinforcing and synergizing each other, we determined six pairs of paradoxical leaders’ behaviors (i.e., strategic thinking—operational thinking, optimism—realism, emotional distance—empathy, rationality—intuitiveness, loose—tight leadership, mobilizing support—providing support) that increase the ability to cope with the crisis, and thus help leaders to navigate their organizations through crisis.

Even though paradoxes are deeply ingrained in the organizational life, especially since today’s leaders are operating in the VUCA world (i.e., volatile, uncertain, complex, and ambiguous; Lewis and Smith 2022), paradoxes in crisis situations are likely to increase as the conditions become more complex, uncertain, and disruptive (e.g., Bundy et al. 2017; Wu et al. 2021). Therefore, crisis situations can be defined as situations where paradoxes are both high and multifarious (e.g., Tabesh and Vera 2020; Carmine et al. 2021; Pradies et al. 2021). While most literature on organizational paradoxes has focused on organizational change (e.g., Carmine et al. 2021; Jay 2013; Luscher and Lewis 2008; Smith and Tracey 2016), we enrich the existing research by showing how leaders respond to organizational crises. This paradoxical approach might be particularly valuable with a view on the COVID-19 pandemic, where organizational paradoxes seem to increase (e.g., Carmine et al. 2021; Pradies et al. 2021), especially since, in order to survive, organizations must maintain a long-term view while being under “incredibly short-term pressure” (Carmine et al. 2021, p. 139). Referring to Tourish’s (2020) editorial article “Why the coronavirus crisis is also a crisis of leadership”, we argue that paradoxical leader’s behavior might be a way of dealing with the paradoxical requirements that are highlighted in the context of the COVID-19 crisis and are, of course, strengthened by the current global developments. With this empirical study, we would like to pave the way for more “theories that explore how leaders can cope with radical uncertainty” (Tourish 2020, p. 265), and how this behavior might help organizations in pursuing a resilient response to these events.

As any study, our study also suffers from limitations. First, we did not include contrast groups, which means that we did not compare our findings with leaders who operate in organizations that eventually file for bankruptcy. We did not do this because we know that there are plenty of factors that also influence the resilient organizational response to crisis, for instance financial slack (e.g., Williams et al. 2017), whereby some factors are out of the leaders’ scope of action (e.g., different department) or have been made by other leaders in the past (e.g., extensive austerity measures). This means that even if the leaders had behaved adequately, the organization could still have gone bankrupt. Treating the leaders’ crisis behavior as the only decisive factor could therefore distort the results. We thus recommend future research, for instance with the help of a quantitative study design, to examine more closely the factors that influence the resilient organizational response to crisis, particularly with regard to the leader’s sphere of influence. Second, we offer a qualitative interview study to explore leaders’ crisis behavior and gain new knowledge in this field. However, our interview results are not validated and, thus, not generalizable. On the one hand, future research in this context might explore more deeply the individual differences of how leaders experience tension and paradoxes during crisis. Following Lewis and Smith (2022), people do not only “differ in the extent to which they experience tension in a situation”, for instance during crisis, “[t]they also differ in their mindset, approaching [this] tension” (Lewis and Smith 2022, p. 13). On the other hand, future studies might expand our results by identifying paradoxes and leaders’ reactions to these paradoxes with the help of a quantitative approach. Third, in our study, we did not distinguish different types of crises and their impact on leaders’ behavior. In this context, event systems theory implies that it is important to consider the characteristics of the event impacting the organization (Morgeson et al. 2015). According to the authors, events in terms of their strength “become more salient when they are novel, disruptive, and critical”, whereby also the event time, for instance in terms of its duration might affect the outcome of the event (Morgeson et al. 2015, p. 515). More recently, Roulet and Bothello (2022, p. 2) further outlined “how certain characteristics among events” led to specific experiences on the individual level, thus focusing on disruptions such as COVID-19 that occur rather from an event chain instead of a single event. Along this line, there are probably different aspects such as the strength, duration but also the temporality and causality of such events that affect the resilient organizational response to crisis, and thus, might also require different leadership approaches.

Since “each type of crisis is unique” including “different threats and challenges” for both the organization as well as the leaders (Wu et al. 2021, p. 17; James et al. 2011), future studies could deepen our initial insights by examining leaders’ crisis behavior in specific types of crises and with different organizational cultures. Crises of different strength and duration might encompass different levels of paradoxes, which in turn requires a different behavior by the leaders. Further, dealing with paradoxical and uncertain situations might differ from one organizational culture to another and has thus different consequences for the resilience of these organization. Along this line, future studies might also include further variables such as the organization’s context, type, or size. Following Bowers et al. (2017), it might also be interesting to examine if leaders’ behavior needs to be different whether it’s an internal or an external crisis. Living in a world that is increasingly characterized by volatility, uncertainty, complexity, ambiguity, and unfortunately crisis, paradoxes do not only “offer a lens to understand organizational phenomena” (Lewis and Smith 2022, p. 22) but also for crisis management and resilience.

Overall, our study provides first insight into how leaders can effectively deal with paradoxes arising in crisis situations and how this behavior might help them foster organizational resilience. We hope that with our empirical study we encourage further researchers to examine the meaning of paradoxes for both leadership and organizational resilience.

## Supplementary Information

Examples from the data in terms of additional data related to the first-order constructs of the analysis

## References

[CR1] Avey JB, Avolio BJ, Luthans F (2011). Experimentally analyzing the impact of leader positivity on follower positivity and performance. The Leadership Quarterly.

[CR2] Bennis W, Thomas RJ (2002). Crucibles of leadership. Harvard Business Review.

[CR3] Best S, Gooderham P (2015). Improvisation: a legitimate strategy in the face of adversity. Small Enterprise Research.

[CR4] Bhamra R, Dani S, Burnard K (2011). Resilience: The concept, a literature review and future directions. International Journal of Production Research.

[CR5] Blanchard KH, Zigarmi D, Nelson RB (1993). Situational Leadership® after 25 years: a retrospective. Journal of Leadership Studies.

[CR7] Boin A, Kuipers S, Overdijk W (2013). Leadership in times of crisis: A framework for assessment. International Review of Public Administration.

[CR8] Borekci D, Rofcanin Y, Sahin M (2014). Effects of organizational culture and organizational resilience over subcontractor riskiness: A multi-method study in longitudinal time setting. European Business Review.

[CR9] Bowers MR, Hall JR, Srinivasan MM (2017). Organizational culture and leadership style: The missing combination for selecting the right leader for effective crisis management. Business horizons.

[CR6] Brockner J, James EH (2008). Toward an understanding of when executives see crisis as opportunity. The Journal of Applied Behavioral Science.

[CR10] Bundy J, Pfarrer MD, Short CE, Coombs WT (2017). Crises and crisis management: Integration, interpretation, and research development. Journal of Management.

[CR11] Buyl T, Boone C, Wade JB (2019). CEO narcissism, risk-taking, and resilience: An empirical analysis in US commercial banks. Journal of Management.

[CR12] Campbell DT, Rock I (1990). Asch’s moral epistemology for socially shared knowledge. The legacy of Solomon Asch: essays in cognition and social psychology.

[CR13] Carmeli A, Friedman Y, Tishler A (2013). Cultivating a resilient top leadership team: The importance of relational connections and strategic decision comprehensiveness. Safety Science.

[CR14] Carmine S, Andriopoulos C, Gotsi M, Härtel CEJ, Krzeminska A, Mafico N, Pradies C, Raza H, Raza-Ullah T, Schrage S, Sharma G, Slawinski N, Stadtler L, Tunarosa A, Winther-Hansen C, Keller J (2021). A paradox approach to organizational tensions during the pandemic crisis. Journal of leadership inquiry.

[CR15] Coutu D (2002). How resilience works. Harvard business review.

[CR16] Duchek S (2014). Growth in the face of crisis: the role of organizational resilience capabilities. Academy of Management Annual Meeting Proceedings.

[CR17] Duchek S (2020). Organizational resilience: a capability-based conceptualization. Business Research.

[CR18] Fietz B, Hillmann J, Guenther E (2021). Cultural effects on organizational resilience: Evidence from the NAFTA Region. Schmalenbach Journal of Business Research.

[CR19] Flanagan JC (1954). The critical incident technique. Psychological Bulletin.

[CR20] Foerster C, Duchek S, Dhiman SK, Marques JF (2022). Leaders’ resilience: What leaders can learn from the COVID-19 crisis. Leadership after COVID-19 working together toward a sustainable future.

[CR21] Ford CM, Gioia DA (2000). Factors influencing creativity in the domain of managerial decision-making. Journal of Management.

[CR22] Gilly J-P, Kechidi M, Talbot D (2014). Resilience of organisations and territories: The role of pivot firms. European Management Journal.

[CR24] Gioia DA, Corley KG, Hamilton AL (2012). Seeking qualitative rigor in inductive research: Notes on the Gioia methodology. Organizational research methods.

[CR23] Gittell JH, Cameron K, Lim S, Rivas V (2006). Relationships, layoffs, and organizational resilience: Airline industry responses to September 11. The Journal of Applied Behavioral Science.

[CR25] Giustiniano L, Cunha MP, Simpson AV, Rego A (2020). Resilient leadership as paradox work: notes from COVID-19. Management and Organization Review.

[CR26] Glaser BG, Strauss AL (1967). The discovery of the grounded theory.

[CR27] Golan N (1978). Treatment in crisis situations.

[CR28] Gooty J, Gavin MB, Johnson P, Frazier ML (2009). In the eyes of the beholder: Transformational leadership, positive psychological capital, and performance. Journal of Leadership & Organizational Studies.

[CR29] Gunderson LH, Holling CS (2001). Panarchy: Understanding transformations in human and natural systems.

[CR30] Harland L, Harrison W, Jones JR, Reiter-Palmon R (2005). Leadership behaviors and subordinate resilience. Journal of Leadership & Organizational Studies.

[CR31] Hartmann S, Weiss M, Newman A, Hoegl M (2020). Resilience in the workplace: A multilevel review and synthesis. Applied Psychology.

[CR32] Hepfer M, Lawrence TB (2022). The heterogeneity of organizational resilience: exploring functional, operational and strategic resilience. Organization Theory.

[CR36] Hersey P, Blanchard K (1988). Management of organizational behavior.

[CR37] Hillmann J, Duchek S, Meyr J, Guenther E (2018). Educating future managers for developing resilient organizations: The role of scenario planning. Journal of Management Education.

[CR38] Horne JF, Orr JE (1998). Assessing behaviors that create resilient organizations. Employment Relations Today.

[CR39] James EH, Wooten LP (2005). Leadership as (un)usual: how to display competence in times of crisis. Organizational Dynamics.

[CR40] James EH, Wooten LP (2010). Leading under pressure: From surviving to thriving before, during, and after a crisis.

[CR2011] James EH, Wooten LP, Dushek K (2011). Crisis management: Informing a newleadership research agenda. Academy of Management Annals.

[CR41] Jay J (2013). Navigating paradox as a mechanism of change and innovation in hybrid organizations. Academy of Management Journal.

[CR42] Kahn WA, Barton MA, Fellows S (2013). Organizational crises and the disturbance of relational systems. Academy of Management Review.

[CR43] Kerrissey, Michaela J., and Amy C. Edmondson. 2020. What good leadership looks like during this pandemic. *Harvard Business Review*. https://hbr.org/2020/04/what-good-leadership-looks-like-during-this-pandemic. Accessed 31 January 2022.

[CR44] King A (1995). Avoiding ecological surprise: Lessons from long-standing communities. Academy of Management Review.

[CR45] Lengnick-Hall CA, Beck TE, Nemeth CP, Hollnagel E, Dekker S (2009). Resilience capacity and strategic agility: Prerequisites for thriving in a dynamic environment. Preparation and restoration.

[CR46] Lengnick-Hall CA, Beck TE, Lengnick-Hall ML (2011). Developing a capacity for organizational resilience through strategic human resource management. Human Resource Management Review.

[CR47] Levinthal DA, March JG (1993). The myopia of learning. Strategic Management Journal.

[CR48] Lewis MW, Smith WK (2022). Reflections on the 2021 decade award: navigating paradox is paradoxical. Academy of Management Review.

[CR49] Li PP (2020). Organizational resilience for a new normal: Balancing the paradox of global interdependence. Management and Organization Review.

[CR50] Limnios EAM, Mazzarol T, Ghadouani A, Schilizzi SGM (2014). The resilience architecture framework: Four organizational archetypes. European Management Journal.

[CR51] Linnenluecke MK (2017). Resilience in business and management research: A review of influential publications and a research agenda. International Journal of Management Reviews.

[CR52] Linnenluecke MK, Griffiths A (2010). Beyond adaptation: resilience for business in light of climate change and weather extremes. Business & Society.

[CR53] Luscher LS, Lewis MW (2008). Organizational change and managerial sensemaking: Working through paradox. Academy of Management Journal.

[CR55] Mafabi S, Munene JC, Ahiauzu A (2015). Creative climate and organisational resilience: the mediating role of innovation. International Journal of Organizational Analysis.

[CR56] Mallak LA (1998). Measuring resilience in health care provider organizations. Health Manpower Management.

[CR57] McCann JE, Selsky JW (2012). Mastering turbulence: the essential capabilities of agile and resilient individuals, teams, and organizations.

[CR58] Meisler G, Vigoda-Gadot E, Drory A, DuBrin AJ (2013). Leadership beyond rationality: Emotional leadership in times of organizational crisis. Handbook of research on crisis leadership in organizations.

[CR59] Milburn TW, Schuler RS, Watman KH (1983). Organizational crisis. Part I: Definition and conceptualization. Human relations.

[CR60] Mitroff II, Pearson CM, Roux-Dufort C (2007). The psychological effects of crises: Deny denial—grieve before a crisis occurs. International handbook of organizational crisis leadership.

[CR61] Morgeson FP, Mitchell TR, Liu D (2015). Event system theory: An event-oriented approach to the organizational sciences. Academy of Management Review.

[CR62] Mumford MD, Tamara LF, Caughron JJ, Byrne CL (2007). Leader cognition in real-world settings: How do leaders think about crises?. The Leadership Quarterly.

[CR33] Nichols, Chris, C. Hayden Shoma, and Chris Tendler. 2020. 4 Behaviors that help leaders manage a crisis. *Harvard Business Review*. https://hbr.org/2020/04/4-behaviors-that-help-leaders-manage-a-crisis. Accessed 31 January 2022.

[CR34] Nooteboom SG, Termeer CJAM (2013). Strategies of complexity leadership in governance systems. International Review of Public Administration.

[CR54] Norman S, Luthans B, Luthans K (2005). The proposed contagion effect of hopeful leaders on the resiliency of employees and organizations. Journal of Leadership &Organizational Studies.

[CR35] Norris WR, Vecchio RPR (1992). Situational leadership theory: A replication. Group & Organization Management.

[CR63] O’Connor GC (1998). Market learning and radical innovation: A cross case comparison of eight radical innovation projects. Journal of Product Innovation Management.

[CR64] Ortiz-de-Mandojana N, Bansal P (2016). The long-term benefits of organizational resilience through sustainable business practices. Strategic Management Journal.

[CR65] Pearson CM, Clair JA (1998). Reframing crisis management. Academy of Management review.

[CR66] Pinkse J, Gasbarro FF (2019). Managing physical impacts of climate change: An attentional perspective on corporate adaptation. Business & Society.

[CR67] Pradies C, Aust I, Bednarek R, Brandl J, Carmine S, Cheal J, Cunha MP, Gaim M, Keegan AJKL, Miron-Spektor E, Nielsen RK, Pouthier V, Sharma G, Sparr JL, Vince R, Keller J (2021). The lived experience of paradox: how individuals navigate tensions during the pandemic crisis. Journal of Management Inquiry.

[CR69] Reeves, Martin, Nikolaus Lang, and Philipp Carlsson-Szlezak. 2020. Lead your business through the coronavirus crisis. *Harvard Business Review*. https://hbr.org/2020/02/lead-your-business-through-the-coronavirus-crisis. Accessed 31 January 2022.

[CR68] Rego A, Sousa F, Marques C, Cunha MP (2012). Authentic leadership promoting employees’ psychological capital and creativity. Journal of Business Research.

[CR70] Riolli L, Savicki V (2003). Information system organizational resilience. Omega.

[CR71] Roberts KH, Bea R (2001). Must accidents happen? Lessons from high-reliability organizations. Academy of Management Perspectives.

[CR72] Rouleau L, Hällgren M, de Rond M (2020). Covid-19 and our understanding of risk, emergencies, and crises. Journal of Management Studies.

[CR73] Roulet TJ, Bothello J (2022). An event-system perspective on disruption: theorizing the pandemic and other discontinuities through historical and fictional accounts of the plague. Academy of Management Review.

[CR74] Roux-Dufort C (2007). Is crisis management (only) a management of exceptions?. Journal of Contingencies and Crisis Management.

[CR76] Sajko M, Boone C, Buyl T (2021). CEO greed, corporate social responsibility, and organizational resilience to systemic shocks. Journal of Management.

[CR75] Samba C, Dusya V, Dejun TK, Maldonado T (2017). Organizational resilience and positive leadership: An integrative framework. Academy of Management Proceedings.

[CR77] Sayegh L, Anthony WP, Perrewé PL (2004). Managerial decision-making under crisis: The role of emotion in an intuitive decision process. Human Resource Management Review.

[CR78] Schlender B, Tetzeli R (2016). Becoming Steve Jobs: the evolution of a reckless upstart into a visionary leader.

[CR79] Shin J, Taylor SM, Seo M-G (2012). Resources for change: the relationships of organizational inducements and psychological resilience to employees’ attitudes and behaviors toward organizational change. Academy of Management Journal.

[CR88] Siebenhaar KU, Köther AK, Alpers GW (2020). Dealing with the COVID-19 infodemic: Distress by information, information avoidance, and compliance with preventive measures. Frontiers in Psychology.

[CR80] Smith WK (2014). Dynamic decision making: A model of senior leaders managing strategic paradoxes. Academy of Management Journal.

[CR81] Smith WK, Lewis MW (2011). Toward a theory of paradox: A dynamic equilibrium model of organizing. Academy of Management Review.

[CR82] Smith WK, Lewis MW (2022). Both/and thinking: Embracing creative tensions to solve your toughest problems.

[CR83] Smith WK, Tracey P (2016). Institutional complexity and paradox theory: Complementarities of competing demands. Strategic Organization.

[CR84] Smith WK, Tushman ML (2005). Managing strategic contradictions: A top leadership model for managing innovation streams. Organization Science.

[CR85] Smith, Melvin, Ellen van Oosten, and Richard E. Boyatzis. 2020. The best leaders balance analytical and emotional intelligence. *Havard Business Review*. https://hbr.org/2020/06/the-best-managers-balance-analytical-and-emotional-intelligence. Accessed 31 January 2022.

[CR86] Somers S (2009). Measuring resilience potential: An adaptive strategy for organizational crisis planning. Journal of Contingencies and Crisis Management.

[CR87] Souitaris V (2001). Strategic influences of technological innovation in Greece. British Journal of Management.

[CR89] Strauss A, Corbin J (1998). Basics of qualitative research: Techniques and procedures for developing grounded theory.

[CR90] Sutcliffe KM, Paine L, Pronovost PJ (2017). Re-examining high reliability: actively organising for safety. BMJ Quality & Safety.

[CR91] Tabesh P, Vera DM (2020). Top managers’ improvisational decision-making in crisis: a paradox perspective. Management Decision.

[CR92] Tourish D (2020). Introduction to the special issue: Why the coronavirus crisis is also a crisis of leadership. Leadership.

[CR93] Vaaler PM, McNamara G (2004). Crisis and competition in expert organizational decision making: Creditrating agencies and their response to turbulence in emerging economies. Organization Science.

[CR95] Van Maanen J (1979). The fact of fiction in organizational ethnography. Administrative Science Quarterly.

[CR94] Van Der Vegt G, Essens P, Wahlström M, George G (2015). Managing risk and resilience. Academy of Management Journal.

[CR96] Vogus TJ, Sutcliffe KM (2007). The impact of safety organizing, trusted leadership, and care pathways on reported medication errors in hospital nursing units. Medical Care.

[CR97] Walumbwa FO, Peterson SJ, Avolio BJ, Hartnell CA (2010). An investigation of the relationships among leader and follower psychological capital, service climate, and job performance. Personnel Psychology.

[CR98] Weick KE (1993). The collapse of sensemaking in organizations: The Mann Gulch disaster. Administrative Science Quarterly.

[CR99] Weick KE, Sutcliffe KM (2001). Managing the unexpected: Assuring high performance in an age of complexity.

[CR100] Wickert C, de Bakker FGA (2018). Pitching for social change: Toward a relational approach to selling and buying social issues. Academy of Management Discoveries.

[CR102] Wiley N (1988). The micro-macro problem in social theory. Sociological Theory.

[CR101] Williams TA, Gruber DA, Sutcliffe KM, Shepherd DA, Yanfei Zaho E (2017). Organizational response to adversity: Fusing crisis leadership and resilience research streams. Academy of Management Annals.

[CR103] Wu LL, Shao B, Newman A, Schwarz G (2021). Crisis leadership: A review and future research agenda. The Leadership Quarterly.

[CR104] Zheng W, Kark R, Meister AL (2018). Paradox versus dilemma mindset: A theory of how women leaders navigate the tensions between agency and communion. The Leadership Quarterly.

