# Supplementary material for: Leading in the Paradoxical World of Crises: How Leaders Navigate Through Crises
Source: Schmalenbach Z Betriebswirtsch Forsch. 2022 Dec 16;74(4):631–57. doi: 10.1007/s41471-022-00147-7 (PMC9755787; doi:10.1007/s41471-022-00147-7)
Supplement: Supplementary file 1 — Examples from the data in terms of additional data related to the first-order constructs of the analysis [file 41471_2022_147_MOESM1_ESM.docx]

**SUPPLEMENTAL MATERIAL**

**Leading in the Paradoxical World of Crises: How Leaders Contribute to Organizational Resilience During Crises**

| **First-Order Constructs** | **Examples from the Data** |
| --- | --- |
| Conceptual analysis | “And then I projected, 800 hours, these are roughly 100 working days you spend in the car. A lot of time to think.” (I15, chief operating officer, consumer goods market, 2008 financial crisis).  “Well, I would say that's 1 or 2 hours [reflection], every now and then and often not in the company as such, but at home, for example, when things are a bit quieter. Then you think more often about what is needed now, how we should do something, how we can classify what we are doing here, etc.” (I8, head of software, banking industry, corruption).  “I deliberately had a day that I didn't spend in [city delated], where I simply looked at the topics once a week, what is necessary, simply.... what is in the textbook, what no one does in practice (...)” (I13, chief executive manager, nationalization). |
| Scenario planning | “There should be always emergency plans in the drawer, just in case the economy starts weakening.” (I9, head of product development, automotive industry, 2008 financial crisis).  „But the question is always that the time, the crisis, is a huge opportunity, because you simply think more deeply for once. And then you become more creative and say, if it doesn't work this way, maybe it works differently. You think about things that you haven't thought of before because you haven't had the need.” (I23, chief executive officer, mechanical engineering, financial crisis 2008).  “And then you become more creative and say, if it doesn't work that way, maybe it can be done differently. About things that you haven't thought of because you haven't had the need to.” (I18, area manager, agricultural equipment, merger). |
| Environmental observing | “We observed the market very closely, the daily news, we monitored the automotive and aircraft industries very closely. They are an indicator, that is how it is.” (I24, B-1 manager, engineering industry, 2008 financial crisis).  “Basically, this circle (model of the 5 phases) makes sense. Not to ignore these warning signs (...) the earlier I react and the earlier I initiate preparatory measures, the easier it is for me to act on point 3 (damage limitation) by minimizing the damage for the individual employee.” (I19, head of sales, agricultural equipment, merger). |
| Immediately reacting | “And then I was called (...) We, all those responsible, went straight to the data center and first tried to find out where the problem occurred.” (I31, internal communication manager, medical technology industry, drop in sales).  “In a normal day, you have ten main topics to solve, but in a crisis, you have forty side stages where you have to solve occurring problems immediately otherwise you do not survive (...).” (I13, chief executive manager, aviation). |
| Data analysis | “Large corporations are, in particular, strongly driven by three key performance indicators: (...) incoming orders, sales and profit. And if the incoming order suddenly drops, you have to worry. And these indicators are usually consistently followed and considered, regardless of whether times are good (...) [you are] in a crisis.” (I3, IT project manager, construction industry, financial crisis 2008).  “What were the causes of the failure? It was too much expansion, too fast expansion, and whenever you expand too fast as a bank, you have risk, structures that don't work.” (I13, chief executive manager, aviation/bank, nationalization).  “We looked at the financial situation of the company and examined it again (...) in order to consider what effects this collapse has had on us, what we have to do now so that we can simply continue to exist on the market. And with an additional project, we simply examined all the orders we had in our books: Where do we stand, where is the current status, how high is the probability that the customer will drop out?" (I3, IT project manager, construction industry, financial crisis 2008). |
| Stay motivated | “I think optimism is always very important. If we complain and say, ‘For God’s sake!’ and everything, it can only get worse.” (I9, head of product development, automotive, financial crisis 2008).  „You just have to be optimistic so far. Somehow it always works again. Even if you don't see yourself out of it, it still works again. Then you sleep on it for a night, and it works.” (I13, chief executive manager, aviation/bank, nationalization). |
| Stay persevere | “You always have to believe that there is a solution. The moment you no longer believe in it, you have lost.” (I13, chief executive manager, aviation/bank, nationalization).  “When you are the last link in the chain somewhere and are so enormously dependent on the links beforehand, that naturally stressed me out a lot. (...) So I found that extremely stressful, where I also had to curb my impatience.” (C18, internal manager, medical devices, product recall).  “There is no crisis that lasts forever.” (I18, Area Manager, agricultural equipment, merger). |
| Accepting the situation | “I have come to terms with the situation (...) and accept life as it is, so that you simply don't make any great demands, accept what life brings.” (I7, project manager, banking industry, corruption).  “Since it's not the first time I've been involved in something like this, I have to admit the first reaction was simply' yeah, that's probably how it is ‘Let's see what happens’.” (I29, project manager, consulting, financial crisis). |
| Sticking to the facts | “In a crisis, you rely much more on what is written […]. Emails suddenly become very important.” (I3, IT project manager, construction industry, financial crisis 2008)  “But it's my job, of course, to keep a cool head, to keep my nerve. It will get better again, and not just because I claim or say so now, but because I also support and argue with figures.” (I23, chief executive officer, mechanical engineering, financial crisis 2008). |
| Stay calm | “[It is important] that you do not run around like a startled bunny in your daily work and constantly have beads of sweat on your forehead, but that you still face this crisis and your daily business with a smile and with a certain calmness.” (I22, managing director, agricultural equipment, merger).  “If you're used to it for a long time or become more and more used to it, the stress level, (.) then things don't shock you anymore, yes.” (I8 head of software, banking industry, corruption).  “(...) That the guys were actually pretty cool. (...) So (...) in the board, I never saw any signs (.) that anyone was losing their nerve. (...) they've all seen these times before” (I17, managing director, agricultural equipment, merger). |
| Stay rational | “You just have to be able to concentrate on, let’s say, the job and just switch off this (…) this soft, interpersonal relationship.” (I7, project manager, banking industry, corruption).  “I am a very pragmatic person. Of course, I am also responsible for communication: "So, I can now lament about the fact that we don't have that. That doesn't get us anywhere. We only lose precious time.” (I26, internal manager, medical devices, product recall). |
| Show hardiness | “Well, I have to say, when I started here, I let go of a lot. But when I noticed that it was burning, I learned to be harder and more consistent, but then you always have to look in-dividually [to determine] when it is worth increasing the pressure.” (I25, area manager, telecommunication, drop in sales).  “So in the uncertainty I certainly tightened the reins more to give a line.” (I15, chief operating officer, consumer good market, market slump). |
| Sticking together | “We don't do anything on our own, with us all managers are always involved.” (I24, B-1 manager, mechanical engineering, financial crisis).  “It is very important that the leaders have a common understanding.” (I18, area manager, agricultural equipment, merger).  “It was also nice when we saw that we had to do it ourselves. Colleagues even came voluntarily and said: ‘Come on, I'll help you. I'll sit with you tonight (...) So everyone helped in their own way’.” (I26, internal manager, medical devices, product recall). |
| Creation of  cohesion | “Whenever employees know each other well and have a way with each other at a personal level, then collaboration in daily operations runs smoothly.” (I3, IT project manager, construction, financial crisis 2008).  “My principle is that there has to be at least one laugh a day, or some kind of fun, or something like that, just to create a good atmosphere.” (I18, Area Manager, agricultural equipment, merger).  “But I think what really helped was to see, um, that everyone did their job very well. (...) But everyone was also extremely committed during the crisis.” (I26, internal manager, medical devices, product recall). |
| Creating trust | “What again is very important (...) is to give this security and this trust to the employees. But that only works if this topic of trust is already at a very good level in the company.” (I30, founder/manager, construction, 2008 financial crisis).  “I feel that today, too, that the team realizes that I'm hitting the rails for them. That's something I also claim for myself, that I have a lot of empathy and can also convey it.” (I15, chief operating officer, consumer good market, market slump). |
| Reassure employees | “What again is very important (...) is to give this security and this trust to the employees. But that only works if this topic of trust is already at a very good level in the company.” (I30, founder/manager, construction, 2008 financial crisis).  “The most difficult thing in this situation, and I must say that I was a decisive factor, was to keep the people calm. That they don't start to collapse, they have an enormous responsibility. And to make it clear that no one will die if this goes wrong.” (I28, project manager, public law, software crisis). |
| Communicate openly | “We informed the employees extremely often. We kept holding conference calls for our 1000 sales employees.” (I15, chief operating officer, consumer goods market, market slump).  “It is important to have very active communication, which means that as soon as something is clear, but not before, you have to pass it on to the staff at certain points so that they know as much as possible.” (I8, head of software, banking industry, corruption).  “So a lot of communication and teamwork is needed in this area (...).” (I8, head of software, bank, corruption). |
| Creating crisis structures | “I think the organizational structure is very important. I think the most important thing is to have a crisis team and to recognize crisis management as a separate discipline in the company.” (I26, internal manager, medical devices, product recall).  “Of course, as a company we have a complete crisis management system. Er, in which it is precisely documented who has to be involved depending on the crisis, who has which er role and responsibility. Um, that's something we have for, ah, for every situation in the company. There are a lot of systems that then take effect. (...) We know exactly who has to be called. These are also employees who have to be available 24/7, 365 days a year.” (I26, internal manager, product recall, medical devices). |
| Experienced-based acting | “That means after 5 years […], I was much more used to [crisis situations], whereas other people were terrified of [them].” (I13, chief executive manager, aviation, nationalization).  “And then (...) there was a (..) special board meeting at which the (...) he was there (...) where we have presented a collection of possible measures (...) The steel industry has already gone through various crises, so that wasn't necessarily something new for us and there is a certain course of action that always takes place.” (I17, managing director, agricultural equipment, merger).  “So and so you generally deal with these, these things in a very structured way, so you don't panic at all or anything, if you have done it so often.” (I8, head of software, bank, corruption). |
| Stay flexible | “Crisis means I need the greatest possible flexibility to adjust to this situation because I am facing a new situation, and new situations or individual situations always require individual measures. This requires the greatest possible flexibility from the entire company, from the management level to the single employee.” (I30, founder/manager, construction, 2008 financial crisis).  “Then you actually throw all the traditional rules overboard. That’s the only way you can survive.” (I13, chief executive manager, aviation/bank, nationalization). |
| Stay open | „With all the weakness that one can show, in the sense of openness and saying 'Guys, I don't know what tomorrow will bring, but we'll go there together’.” (I15, chief operating officer, consumer good market, market slump).  “But there is always rule and there are always ways that may not be visible first and that is what we have to accomplish here” (I18, area manager, agricultural equipment, merger). |
| Give clear directions | “The most important thing is to provide employees with a clear goal and to explain why they are in a stressful situation and why it is important that they act in a certain way.” (I8, head of software, bank, corruption).  “And then I started to bring the team together once a week, ah I'm more a friend of quick meetings, which we still do once a week on Wednesdays at 9, everybody knows, and we don't sit down, we stand. And that's quick information and it's about strategic things (...)” (I13, chief executive manager, aviation/bank, nationalization). |
| Set clear priorities | “Then you have to set very clear priorities, to say very clearly, ‘I’ll do that now and I can’t do the other thing now.’” (I8, head of software, bank, corruption).  “But it is important for us to say that we have really defined priorities that everyone can believe in today. And everyone has the same picture in their minds.” (I15, chief operating officer, consumer good markets, market slump). |
| Delegate | “You trigger [your employees] a little bit, and then it goes off, and two days later the solution is there, and my input is not needed at all.” (I11, head of legal services, bank, 2008 financial crisis).  “So I think (.) still, a manager will delegate tasks even in a crisis (...) then I also have to define a team, yes, so these are usually employees who are from a close environment of the manager, who are trusted (.) and who then get this task.” (I3, IT-Project Manager, construction, financial crisis).  “(...) and you have to clearly address the tasks and timelines and then you give usually a maximum of one day, so not (...) last second, but (...) they usually only have one or half a day to simulate all the performance data sets and do things and then you have everything together.” (I8, head of software, bank, corruption). |
| Give room for participation | “So, for example, I included people that I absolutely trust. After two weeks, we got along so well that I knew I didn’t need to set a framework.” (I13, chief executive manager, aviation/bank, nationalization).  “The involvement of the employees (...) [we] gave them the feeling that they were in-volved in the process and that is a change process.” (I27, human resource manager, financial crisis 2008). |
| Cooperation with competitors or other firms of the value chain | “[It is important] to build a relationship with competitors, where you are not seen as a competitor but as a market participant (..) who has his core competencies in these are-as but is also ready, if it is not his core competency, to pass service to the best possible company.” (I6, controller, technology, financial crisis 2008).  “It is important not only to think in terms of competition, but above all to look at the entire value chain in the future, i.e., to build up a stable relationship with the suppliers.” (I6, controller, technology, financial crisis 2008). |
| Work with  consultants | “We deliberately looked for people from outside [the company].” (I13, chief executive manager, aviation/bank, nationalization).  „We deliberately brought in a HR manager (I15, chief operating officer, Consumer goods market, market slump).  “And we did a planning process with external consultants (...) who analyzed the whole thing and also looked at where there was potential for improvement, where it could be optimized.” (I18, area manager, agricultural equipment, merger). |
| Giving support | “There is still an offer (...) that you can use leadership coaching for yourself. There is also the possibility [for the employees] to do an anonymous coaching.” (I4, head of human resources, bank, corruption).  “We supported them, we introduced our own health program [name of the health program] (...) Because in a crisis, when everyone is depressed and has fears and insecurity and so on (...), the greatest good is health, so that you are not harmed.” (I13, chief executive officer, aviation/bank, nationalization). |
